# Premature Burial

**Published:** 1910-07-16

**Authors:** 


*To the Editor of* The Hospital.

### PREMATURE BURIAL.

*To the Editor of* The Hospital.

Sir,—Having regard to the overwhelming proofs of the dangers of live sepulture shown by Mr. Basil Tozer's article on the subject in the *Nineteenth Century and After*, referred to by "Live and Let Live" in The Hospital of July 9, and the urgent necessity for reform, I venture, with your kind permission, earnestly to appeal to your readers and the public generally to support the Association for the Prevention of Premature Burial in its endeavours to obtain legal safeguards against the possibilities of such terrible occurrences in this country.

Numerous as are the facts cited by the author of the article alluded to, many indisputable and some recent cases of narrow escapes are omitted. For example, at Hinckley, in Leicestershire, in 1906, a living child was medically certified to be dead and its death registered. Under the title of "Dead Baby Comes to Life" the *Brighton Herald* of May 20, 1905, reported that a local medical man informed the coroner's officer of the death of a child whom he had been attending. An hour or so after the notification the officer called at the house for the purpose of arranging for the removal of the medically certified dead baby to the mortuary, but on uncovering the body, and noticing the life-like appearance of the face, the coroner's officer placed his hand on it, when, to his amazement, the child's eyes opened. The news was immediately conveyed to the astonished mother, and the officer did what was necessary to bring about complete recovery. The child got much better, but had a relapse and died the following day. There is also the case of a Shoreditch man who, on making an application to the magistrate at Old Street Police Court on July 28, 1906, stated that he had been picked up unconscious in the street and taken to the hospital, from whence he was conveyed to a workhouse infirmary. There he was pronounced dead, and laid in a coffin for three days in the mortuary. In a letter to the present writer a correspondent says: "I have an absolute horror of being buried alive, particularly as we have had two cases of the sort in my family; happily, they were discovered before the coffins were closed." Referring to the statement of a correspondent that "everybody should be carefully examined by a doctor before the death certificate is given," Mrs. Annie Reichardt, 54 Carminia Road, Balham, thus relates in the *Norwood News* of June 25 her remarkably narrow escape from interment alive: "I beg to relate my own experience. It was in the early sixties in Cairo, Egypt. I was under thirty. My own regular doctor, Patterson by name, was at my side; and at that time a well-known specialist from Alexandria, Ogilvie by name, was in the house. 'All is over; every sign of death is there,' said the doctor. The pulse was gone; the heart was still; breath entirely ceased; extremities icy cold. At last the eyelids were raised and the pupils examined. 'They have not yet broken, but that will soon follow now,' said the doctor. Two English lady friends were there, and with the two doctors accompanied my husband to the drawing-room to make arrangements for the funeral, which was to take place late the same evening or at sunrise the next morning. The body was left alone for from half to three quarters of an hour. I was perfectly conscious and had heard every word distinctly, but could make no sign. Before leaving the house the doctor came up for a final glance at the body. All at once I felt a violent commotion inside of me. I felt my heart throb, I could open my eyes and speak, and as the doctor came to the side of my bed I said, 'Doctor, I am not going to die now; I feel better.' He started back for a moment, and then took my wrist to feel my pulse; then, slowly and hesitatingly, as if he could not credit it, said, half to himself, 'Yes, it is true; the pulse is beating again. I never saw anything like this before.'"

Numbers of similar narrow escapes might be quoted, and it is probable that for one discovery a hundred death-counterfeits escape detection.

I am, Sir, yours respectfully,

July 11. "Waiting Mortuary."

